# Inhibition of DEC2 is necessary for exiting cell dormancy in salivary adenoid cystic carcinoma

**DOI:** 10.1186/s13046-021-01956-0

**Published:** 2021-05-14

**Authors:** Xiao Yang, Jia-shun Wu, Mao Li, Wei-long Zhang, Xiao-lei Gao, Hao-fan Wang, Xiang-hua Yu, Xin Pang, Mei Zhang, Xin-hua Liang, Ya-ling Tang

**Affiliations:** 1 State Key Laboratory of Oral Diseases & National Clinical Research Center for Oral Diseases & Department of Oral Pathology, West China Hospital of Stomatology (Sichuan University), No.14, Sec. 3, Renminnan Road, Chengdu, 610041 China; 2 Department of Stomatology, Affiliated Hospital of Jining Medical University, Jining Medical University, Jining, China; 3 State Key Laboratory of Oral Diseases & National Clinical Research Center for Oral Diseases & Department of Oral and Maxillofacial Surgery, West China Hospital of Stomatology (Sichuan University), No.14, Sec. 3, Renminnan Road, Chengdu, 610041 China

**Keywords:** DEC2, Salivary adenoid cystic carcinoma, Hypoxia microenvironment, Tumor dormancy, Metastasis, EMT

## Abstract

**Background:**

Patients were prone to have poor prognosis once dormant tumor cells being reactivated. However, the molecular mechanism of tumor cell dormancy remains poorly understood. This study aimed to investigate the function of DEC2 in the dormancy of salivary adenoid cystic carcinoma (SACC) in vitro and vivo.

**Methods:**

The function of DEC2 in tumor dormancy of SACC was investigated in nude mice by establishing primary and lung metastasis model. Meanwhile, the interaction between hypoxia and SACC dormancy and the role of DEC2 were demonstrated through CoCl_2_ induced hypoxia–mimicking microenvironments. Furthermore, the expression of DEC2 was detected by immunohistochemical staining in primary SACC samples with and without recurrence.

**Results:**

In the primary SACC, DEC2 overexpression inhibited cell proliferation, increased cell population arrested in G0/G1 phase, and participated in dormancy regulation, which limited tumor growth. Intriguingly, in the model of lung metastasis, the level of DEC2 was reduced significantly and resulted in dormancy exit and growth resumption of SACC cells. Then, we found that DEC2 may associate with hypoxia in contributing to tumor dormancy, which might provide a possible cue to explain the different roles of DEC2 in primary and metastasis lesions. And overexpression of DEC2 induced dormancy and promoted migration and invasion through activating EMT program. Finally, DEC2 positive expression was shown to be significantly correlated with recurrence and dormancy of SACC patients.

**Conclusions:**

These findings provide a novel insight into the role of DEC2 gene in tumor dormancy and metastasis.

**Supplementary Information:**

The online version contains supplementary material available at 10.1186/s13046-021-01956-0.

## Background

Salivary adenoid cystic carcinoma (SACC) is one of the most common malignancies characterized with slow growth, but high incidence of local recurrence and metastasis [1, 2]. Although the 5-year disease-free survival rate generally reaches 90%, it drops to 10% after 20 years owing to potential local recurrence and hematogenous distant metastases [3]. Therefore, it is necessary to elucidate the molecular mechanisms of recurrence and metastasis, which will provide molecular targets for the treatment of SACC patients.

Tumor dormancy was a period in tumor progression in which residual disease existed but remained asymptomatic clinically for years or even decades [4]. It may appear during the formation of primary tumor, after dissemination of primary tumor cells or in the micrometastasis [5, 6]. Tumor cell dormancy is defined at cellular level which is characterized with cells that are not divided and arrested in G0/G1 cell phase [7]. The dormant tumor cells could escape immune surveillance and chemo-radiotherapies, remaining undetectable for long periods [6]. Dormant tumor cells are emerging as a critical cause for recurrence and metastasis once they escape this state [8]. However, how tumor cells enter into dormancy and what processes govern their exit in human cancers including SACC are still absent and remain unclear.

Differentiated embryonic chondrocyte gene 2 (DEC2, also known as BHLHE41/BHLHB3/Sharp1) is one of the basic helix-loop-helix (bHLH) transcription regulators, which has been demonstrated to play important roles in regulating circadian rhythms, cell proliferation, hypoxia reaction, immune responses as well as malignant tumor progression [9–12]. Recent evidence demonstrated that inhibition of DEC2 and nuclear receptor subfamily 2 group F member 1(NR2F1) resulted in increased growth of breast cancer cells [13] and downregulation of DEC2 interrupted tumor cell dormancy [14], indicating DEC2 might involve in tumor dormancy. And TGF-β2 activated a [ERK/p38]^low^ signaling ratio, which resulted in induction of DEC2 and dormancy of malignant disseminated tumor cells (DTCs) in the bone marrow of head and neck squamous cell carcinoma (HNSCC). However, the role and its molecular mechanism of DEC2 in the dormancy and malignant progression of SACC remain unclear.

Here, we found that DEC2 induced tumor dormancy of the primary SACC and in the model of lung metastasis, DEC2 positive tumor cells manifested enhanced migration and invasion and formed more metastases and the level of DEC2 was reduced significantly with the resumption of cell proliferation. Then, DEC2 may associate with HIF1α in contributing to tumor dormancy, which might provide a possible cue to explain the different roles of DEC2 in primary and metastasis lesions. Our findings demonstrated that overexpression of DEC2 contributed to the dormancy of tumor and low expression reawakened cell dormancy of SACC, which may provide important implications for the therapy of patients.

## Materials and methods

### Xenografts

Lentivirus-transfected SACC-83 cells were subcutaneously injected s.c. (5 × 10^6^cells/200 μL PBS/mouse) into the flank of 6-week-old nude female mice (Laboratory Animal Center of Sichuan University, Chengdu, China) and examined every 3 to 5 days for tumor appearance. Tumor growth was then measured once a week until 35 days after inoculation by determining the tumor volumes using caliper measurements. Lentivirus-transfected SACC-83 cells (5 × 10^6^cells/200 μL PBS/mouse) were injected via tail vein of nude female mice to establish the model of lung metastasis. The nude mice were weighed weekly. Lung tissues were excised after 4 weeks for immumohistochemical staining. All animal experiments were approved by the Institutional Ethics Committee of the West China Medical Center, Sichuan University, China.

### Immunohistochemistry

Paraffin-embedded sections were cut into 4um and deparaffinized in xylene and rehydrated, and endogenous peroxidase was blocked with 3%H_2_O_2_. Antigen retrieval was accomplished by 0.01 mol/L citrate buffer solution (pH 6.0) in a 700 W microwave oven for 15 min. After incubation with 5% normal goat serum for 20 min, the slides were exposed overnight at 4 °C to the rabbit anti-DEC2 (1:150; Proteintech), rabbit anti-Ki-67(1:800; Proteintech), rabbit anti-NR2F1 (1:200; Proteintech). Sections were then incubated with biotinylated goat anti-rabbit IgG (Zhongshan Goldenbridge Biotechnology) for 1 h, and streptavidin-peroxidase for 30 min. The 0.02% diaminobenzidine tetrahydrochloride was used as a chromogen, and the slides were counterstained with hematoxylin. The percentage of positive cells was estimated using an image analysis system (Leica).

### Cell lines and cell culture

Two SACC cell lines, SACC-83 and SACC-LM, were obtained from the State Key Laboratory of Oral Disease, Sichuan University. Cells were cultured in RPMI 1640 medium (Gibco) supplemented with 10% heat-inactivated FCS (Hyclone), 2 mmol/L L-glutamine, 25 mmol/L HEPES, and 100 units/mL penicillin and streptomycin at 37°Cin 5% CO_2_. For hypoxic treatment, cells were exposed to 0.1% O_2_ with 5% CO_2_ at 37 °C with hypoxia chamber.

### Cloning, Lentivirus preparation, and plasmids

The targeted cDNA of DEC2 was cloned into the pEZ-Lv201 and constructed into EX-W0115-Lv201 plasmid and negative control plasmid EX-NEG-LV201. After sequencing verification, it was packaged into virus. Human DEC2, BC_025968, total 1449 bp. P/Puro plasmid vector and transfected into cells by Lipofectamine 2000 (Invitrogen) according to the manufacturer’s instructions. The stable transfected cells were selected with puromycin.

### Transient siRNA knockdowns

SiRNAs targeting DEC2 and Slug and their control siRNAs were purchased from Genechem. The target sequences were as following: DEC2 siRNA-1: CUCCCUAUAUCCCAAUGGATT, UCCAUUGGGAUAUAGGGAGTT; DEC2 siRNA-2: CGAGGAAGAACUAUGAACATT, UGUUCAUAGUUCUUCCUCGTT;

DEC2 siRNA-3: GAUGAAAGAAUUACCGAAUTT; AUUCGGUAAUUCUUUCA UCTT; Control siRNA CUCUCCGAACGUGUCACGUTT; GCGUGACACGUUCG GAGAATT. The above transient transfections in SACC cells were performed using 20 μM of each siRNA with Lipofectamine 2000 (Invitrogen, Carlsbad, CA, USA). Knockdown was verified by qRT-PCR and Western Blot.

### Quantitative real-time RT-PCR

Total RNA was extracted from cells using the trizol (Invitrogen, Carlsbad, CA) and were quantified with the NanoDrop ND-1000 Spectrophotometer (Thermo ScientificInc., Waltham, MA). PCR amplification of the cDNA template was done using Thunderbird SYBR qPCR mix (TOYOBO) on ABI PRISM 7300 sequence detection system (Applied Biosystems) according to the manufacturer’s protocol. The resulting cDNA was diluted and used as a template for Quantitative real-time PCR using LightCycler (Roche Diagnostics GmbH, Mannheim, Germany). β-actin was used as the housekeeping gene to normalize the target gene expression. The sequences of PCR primers were showed in Supplementary Table S1.

### Western blot

Total proteins were extracted from the cultured cells with a total protein extraction kit (Keygen, Nanjing, China). The protein concentrations were detected by a BCA Protein Assay Kit (Beyotime, Shanghai, China). Protein samples were then separated by 6 and 8% sodium dodecyl sulfate–polyacrylamidegel electrophoresis (SDS-PAGE) and blotted on polyvinylidenefluoride (PVDF) membranes. Membranes were blocked in phosphate-buffered saline/Tween-20 containing 5% non-fat milk and incubated with the following primary antibodies: DEC2 (Proteintech Group, Chicago, USA), HIF-1α (Wanleibio, China), Slug (Proteintech Group, Chicago, USA), Snail (Proteintech Group, Chicago, USA), β-actin (Sigma-Aldrich). Horse radish peroxidase–conjugated anti-rabbit or anti-mouse IgG were used as the secondary antibody (TA322704 or TA326473, ZSGB-BIO, China, 1:1000). Subsequent visualization was detected using a densitometer (GS-700, Bio-Rad Laboratories).

### Immunofluorescence staining

SACC cells were cultured in 12-well cell culture plates. Upon reaching 70% confluence, cells were washed in cold PBS and fixed in 4% paraformaldehyde for 30 min, permeabilized in 0.25% Triton X-100 in PBS for 15 min, and blocked with 1% bovine serum albumin prepared in PBS for 30 min. Lastly, cells were incubated overnight with mouse anti-Ki-67 (1:100 dilution), and then incubated with FITC or TRITC-conjugated goat anti-mouse IgG (1:500; Zhongshan Goldenbridge Biotechnology) at 37 °C for 1 h. Cells were visualized using the Olympus Fluoview confocal microscope (Tokyo, Japan), and fluorescence images were taken.

### Wound healing assay

SACC cells were seeded and cultured in 6-well plates. Upon reaching 80% confluence, cells were wounded by scratching with a pipette tip and incubated with medium containing no FBS for 24 h. Then, they were photographed under phase-contrast microscopy.

### In vitro cell invasion assay

Invasion of cells was assessed using Matrigel-coated membrane (24-well insert, pore size, 8 μm; BD Biosciences). About 5 × 10^4^ cells were plated in the top chamber in serum free medium, and medium with serum was used as a chemo-attractant in the lower chamber. After 48 h of incubation, cells remaining on the top chamber were removed using a cotton swab. Traversed cells on the lower surface of the membrane were fixed in 4% paraformaldehyde and stained with 1% Crystal Violet; five fields per filter were counted.

### Cell proliferation assays

The cell proliferation was assessed by Cell Counting Kit-8 (CCK-8, Dojindo) assay. Cells were seeded in 96-well plates in triplicate and the proliferation assay was performed after 24 h incubation. 10ul of CCK-8 solution was added into per well and the absorbance reading was measured at 450 nm after 30 min of incubation at 37 °C. The above experiments were repeated the next 4 days.

### Apoptosis detection by FCM

Cell apoptosis was performed by combined application of Annexin V–FITC and propidium iodide (BD Biosciences Clontech, USA). Cells were washed by PBS and adjusted to1 × 10^6^ cells/ml with 4 °C PBS. One hundred microliter of suspensions was added to each labeled Falcon tube (12 mm × 75 mm, polystyrene round-bottom); 10 μl of Annexin V–FITC and 10 μl propidium iodide (20 μg/ml) were added into the above labeled tube, which was incubated for 30 min at room temperature in the dark environment; and then 400 μl PBS binding buffer was added to each tube which was analyzed using FCM analysis (BD Biosciences Clontech, USA).

### Glucose consumption test

Glucose consumption was detected using a glucose assay kit (Nobio, China). About 1 × 10^5^ cells/well was seeded in 6-well plates. The test was performed according to the manufacturer’s protocol. The experiments were performed at least three times.

### Cell senescence detection

Senescent cells were measured using a senescence β-galactosidase staining kit (Beyotime, China). Cells were seeded in 6-well plates (1 × 10^5^ cells/well). The staining was performed according to the manufacturer’s instructions. The cells were then observed under an Olympus BX51 microscope and were analyzed using ImageJ software.

### Clinical samples collection and study

The cohort was assembled from 70 patients who were histologically diagnosed with SACC and underwent resection of their tumors at West China Hospital of Stomatology, Sichuan University, between 2005 and 2015. Exclusion criteria included preoperative chemotherapy, hormone therapy or radiotherapy. All samples of SACC were collected at the time of surgery. All the paraffin-embedded sections were confirmed histologically with blind method by two pathologists. The protocol of the study was approved by the Institutional Ethics Committee of the West China Medical Center, Sichuan University, China. The pathologic characteristics of the tumors and clinical data of the patients were summarized in Table 1.

**Table 1:** Clinical-pathologic characteristic of 70 patients with SACC, and the association between DEC2 expression and these variables

Clinical-pathologic variables		DEC2 (nuclear)			
		No. of patients	Negative	Positive	*P*
Age (years)	≤55	34	25	9	0.684
	>55	36	28	8	
Gender	Male	31	23	8	0.795
	Female	39	30	9	
Tumor site	Major salivary gland	21	18	3	**0.043**
	Minor salivary gland	49	30	9	**8**
T stage	T1/T2	25	16	9	0.091
	T3/T4	45	37	8	
Histological subtype	Cribriform/Tubular	58	38	20	**0.009**
	Solid	12	3	9	
Recurrence	With	33	20	13	**0.005**
	Without	37	33	4	
metastasis	With	31	18	13	**0.002**
	Without	39	35	4	

Immunohistochemistry results showed that the nuclear staining of DEC2 was detected in 17 of 70 and it was significantly associated with tumor site, histological subtype, recurrence and metastasis. Neither was related to age, gender or T stage

### TUNEL assay

Terminal deoxynucleotidyl transferase-mediated dUTP nick and labeling (TUNEL) Kit (KeyGEN) was to test cell apoptosis. Negative was graded as 0 to 10% within 4–6 microscopic fields at × 400 magnification; and the positive was graded as more than 10% as well.

### Statistical analyses

All statistical analyses were conducted using GraphPad Prism. Data was plotted with GraphPad Prism software. A value of *P* < 0.05 was considered statistically significant. All experiments were performed independently at least three times.

## Results

### High expression of DEC2 induced tumor dormancy of the primary SACC in nude mice

**Fig. 1:**
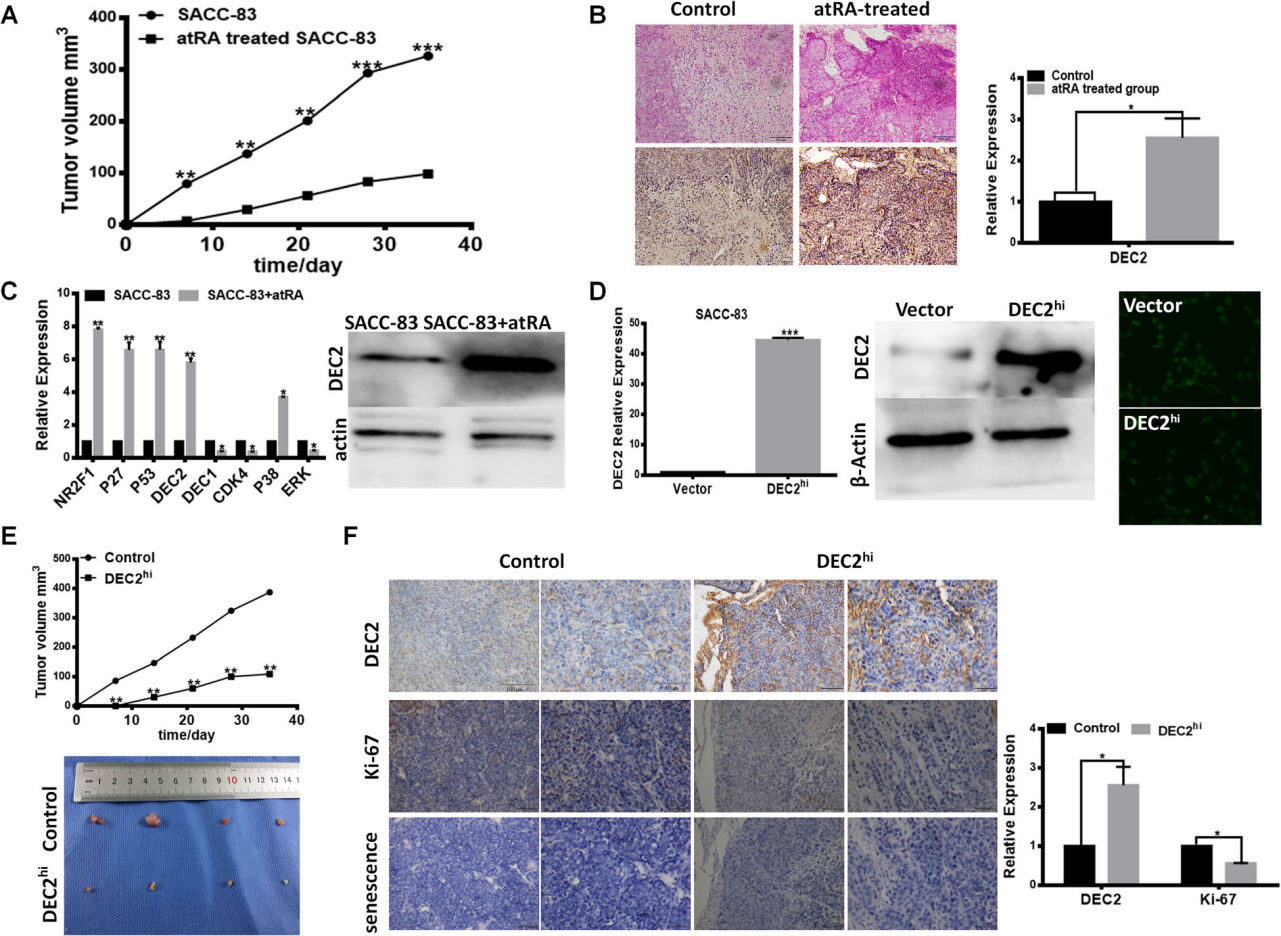
High expression of DEC2 induced tumor dormancy of the primary SACC in nude mice. **a** and **b** Tumor volume (**a**) and immunohistochemical staining of DEC2 (**b)** in atRA treatment and control group. **c** Fold change in mRNA expression of NR2F1, P27, P53, DEC2, CDK4, P38 and ERK, and the protein expression of DEC2 after atRA treatment compared to control group. **d** DEC2 over expression was confirmed by QPCR, WB and immunofluorescence in SACC-83 cells. **e** and **f** SACC-83 cells with or without DEC2 overexpression were injected into nude mice subcutaneously to observe tumor development. Tumor volumes were determined (**e**) and the expression of DEC2, HIF1α and Ki-67 were detected by immunohistochemical staining (**f**). Error bars represent the mean ± SD of triplicate experiments. (*P < 0 .05, **P < 0.01, ***P < 0 .001)

Our previous findings have demonstrated that atRA treatment can be used to induce dormancy in SACC cells, and its effects on cancer dormancy resulted directly from modulation of NR2F1 [15, 16]. To further examine the molecular mechanism of tumor dormancy of SACC, atRA treated SACC-83 and the control cells were injected subcutaneously into nude mice, respectively. As shown in Fig. 1a, tumor volume in atRA treated group was much smaller than the control group after subcutaneously injecting. Interestingly, immunohistochemistrical staining showed the positive expression of DEC2 in the xenograft of atRA treated group and the negative expression in the control group (Fig. 1b). The data showed that upregulation of DEC2 involved in atRA treatment contributing to tumor dormancy of the xenograft model. We subsequently investigated the expression of DEC2 in the dormant state induced by atRA in SACC cells by qRT-PCR and Western blot. The results demonstrated that DEC2 was also involved in the dormant state induced by atRA besides NR2F1 (Fig. 1c).

To address the different roles of DEC2 in the primary lesion and metastasis location of SACC nude mice, we stably overexpressed DEC2 in SACC-83 cells by using lentivirus infection, as confirmed by real-time PCR, WB and immunofluorescence *(*Fig. 1d). DEC2 overexpression and vector SACC-83 cells were injected respectively into nude mice. As shown in Fig. 1e, DEC2 overexpressed groups exerted a slower tumor growth than the vector groups. And tumor volume in DEC2-overexpressed group of the xenograft nude mice model was smaller than the vector in 5 weeks after subcutaneously injecting SACC-83 cells. This indicated that DEC2 might induce dormancy and inhibit tumor growth of the primary SACC. Immunohistochemistrical staining showed the positive expression of DEC2 and weak positive expression of Ki-67 in the xenograft of DEC2-overexpressed group. And the vector group displayed high expression of Ki-67 (Fig. 1f). These results suggested that DEC2 overexpression contributed to tumor dormancy of the primary SACC.

### Low level of DEC2 exited dormancy to promote lung metastasis of SACC in nude mice

**Fig. 2:**
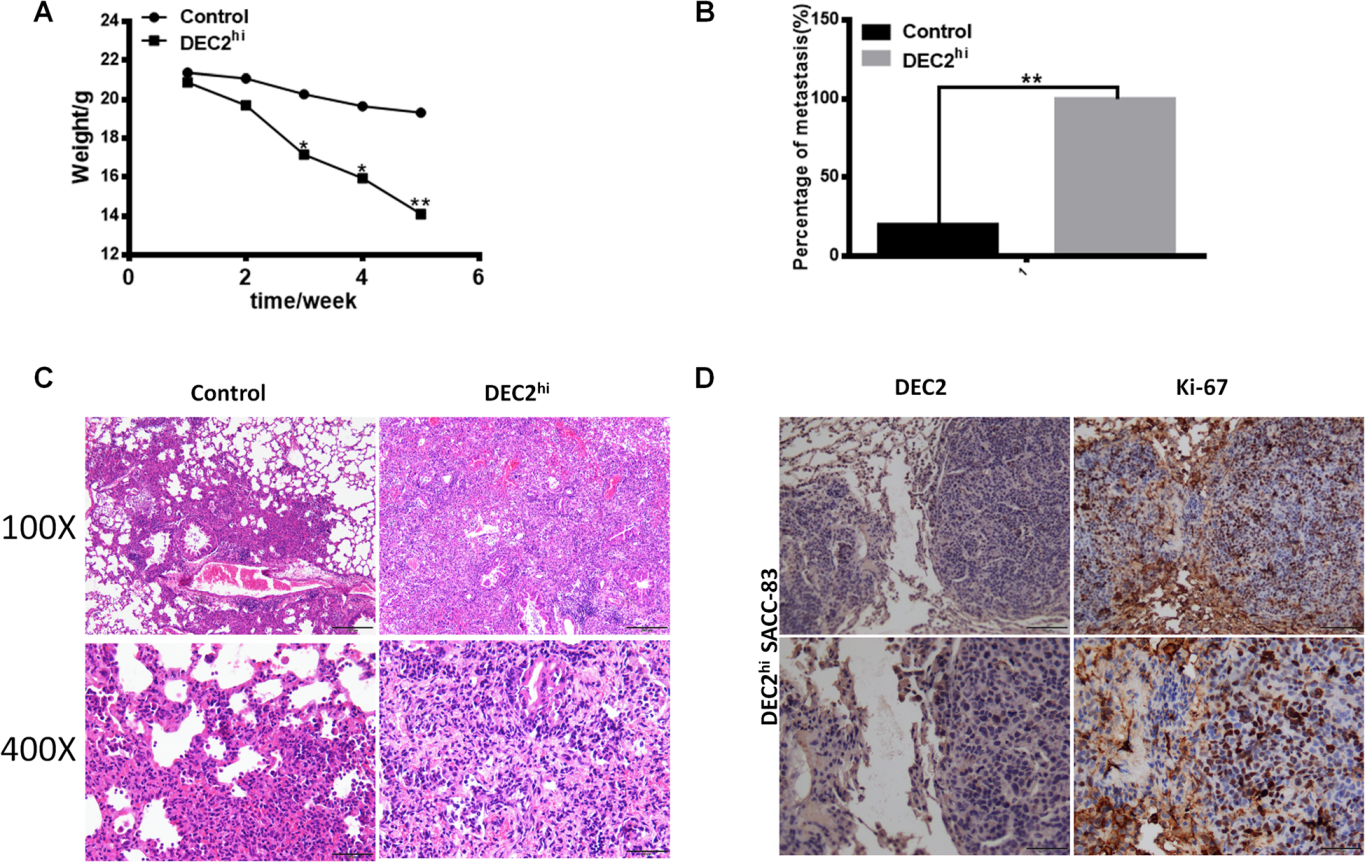
Low level of DEC2 exited dormancy to promote lung metastasis of SACC in nude mice. **a** The weight of nude mice between DEC2-overexpression and vector cell group in lung metastasis model. **b** Comparison of lung metastasis rate between DEC2-overexpression and vector cell group. **c** HE staining of lung metastasis of the two groups. **d** The expression of DEC2 and Ki-67 were determined by immunohistochemical staining in DEC2 overexpressed group. Error bars represent the mean ± SD of triplicate experiments. (*P < 0 .05, **P < 0.01, ***P < 0 .001)

We next established lung metastasis model in nude mice by injecting DEC2 overexpression and vector SACC-83 cells into tail vein. As shown in Fig. 2a, the weight of nude mice in DEC2-overexpressed group was significantly lower than the vector group. Only 20% (1/5) of nude mice implanted with vector cells produced lung metastasis, and 100% (5/5) of the mice injected by DEC2 overexpressed cells developed lung metastases (Fig. 2b), indicating that DEC2 expression promoted metastasis of SACC to the target organ of lung tissue. HE staining confirmed the tumor metastatic lumps in lung tissues of DEC2 overexpressed group (Fig. 2c). Then how did the dormant SACC cells with DEC2 overexpression form more metastases in lung of nude mice? We hypothesized that these dormant tumor cells were reactivated into proliferative ones by the oxygen-rich environment in the lung tissue accompanied by downregulation of DEC2. So we further explored the expression of DEC2 by immunohistochemical staining and found the positive expression of Ki-67, while the negative expression of DEC2 in the metastases (Fig. 2d). This confirmed that DEC2 promoted lung metastasis of SACC in nude mice, and these dormant tumor cells resumed proliferation with downregulation of DEC2, leading to the occurrence of metastases.

### High expression of DEC2 induced dormancy and promoted migratory and invasive abilities of SACC cells

**Fig. 3:**
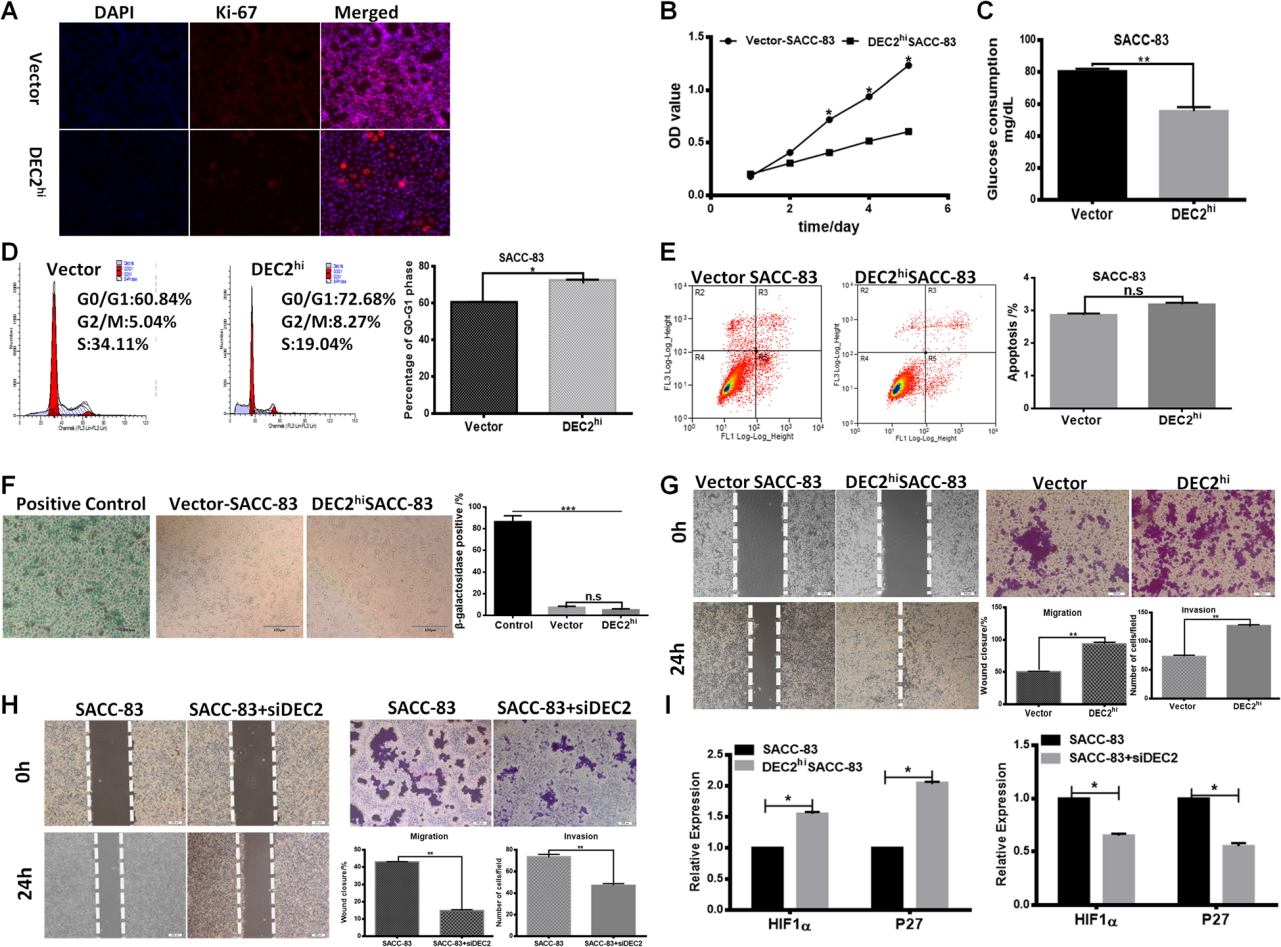
High expression of DEC2 induced dormancy and promoted migratory and invasive abilities of SACC cells. **a-d** The differences of Ki-67 expression (**a**), proliferation capacity (**b**), metabolism (**c**) and cell cycle (**d**) between DEC2 over-expressed and vector SACC-83 cells was detected by immunofluorescence, CCK-8 assay, glucose consumption and Flow cytometry, respectively. **e** and **f** Cell apoptosis test (**e**) and senescent analysis (**f**) in both DEC2 over-expressed and vector cells. **g** and **h** Migration and invasion abilities of DEC2 over-expressed/vector SACC-83 cells (**g**) and SACC-83/SACC-83 + siDEC2 cells (**h**) were detected by transwell and scratch assays, respectively. **i** The mRNA levels of HIF1α and P27 in DEC2 overexpression and silence SACC-83 cells were tested by QPCR. Error bars represent the mean ± SD of triplicate experiments. (*P < 0.05, **P < 0.01, ***P < 0 .001)

We further examined the function of DEC2 in vitro and found that cell proliferation was inhibited after DEC2 overexpression according to immunofluorescence staining of Ki-67 and CCK-8 assays *(*Fig. 3a and b*).*This change in proliferation was confirmed by metabolic capacity, which showed that glucose consumption of DEC2 overexpressed SACC-83 cells was much lower than the control group *(*Fig. 3c*).* Moreover, we tested cell cycle by flow cytometry analysis and found that DEC2 overexpression increased cell population arrested in G0/G1 phase in SACC-83 cells *(*Fig. 3d*)*. Meanwhile, no significant difference of cell apoptosis and senescence were observed between DEC2 overexpressed SACC-83 cells and the control group *(*Fig. 3e and f*)*. And we got similar results in SACC-LM cells (Additional file 1*:* Fig. S1A-C). We next silenced DEC2 using siRNA in SACC cells and the silence efficiency of DEC2 was verified by mRNA and protein expression patterns in Additional file 1*:* Fig. S1D. And it was demonstrated that knockdown of DEC2 promoted tumor cells proliferation and glucose consumption (Additional file 1*:* Fig. S1E-F). The above results indicated that overexpression of DEC2 could inhibit proliferation and induce dormancy of SACC cells.

Then, we detected the effect of DEC2 on the migration and invasion of SACC cells by wound-healing and transwell invasion assays. As shown in Fig. 3g, DEC2 overexpressed SACC cells had higher migratory and invasive behaviors than SACC vector. And knockdown of DEC2 in SACC cells prominently impaired the migration and invasion ability of tumor cells *(*Fig. 3h*)*. The results suggested that DEC2 overexpression dramatically increased the migratory and invasive behaviors of SACC cells.

We next explored the expression of HIF1α and P27 in DEC2 overexpressed SACC-83 cells and found that the mRNA levels of HIF1α and P27 were all increased. In contrast, they were both decreased in DEC2 silenced SACC-83 cells (Fig. 3i). These results indicated that DEC2 may associate with HIF1α in contributing to tumor dormancy, which might provide a possible cue to explain the different roles of DEC2 in primary and metastasis lesions.

### High expression of DEC2 was necessary for CoCl_2_- induced dormancy

**Fig. 4:**
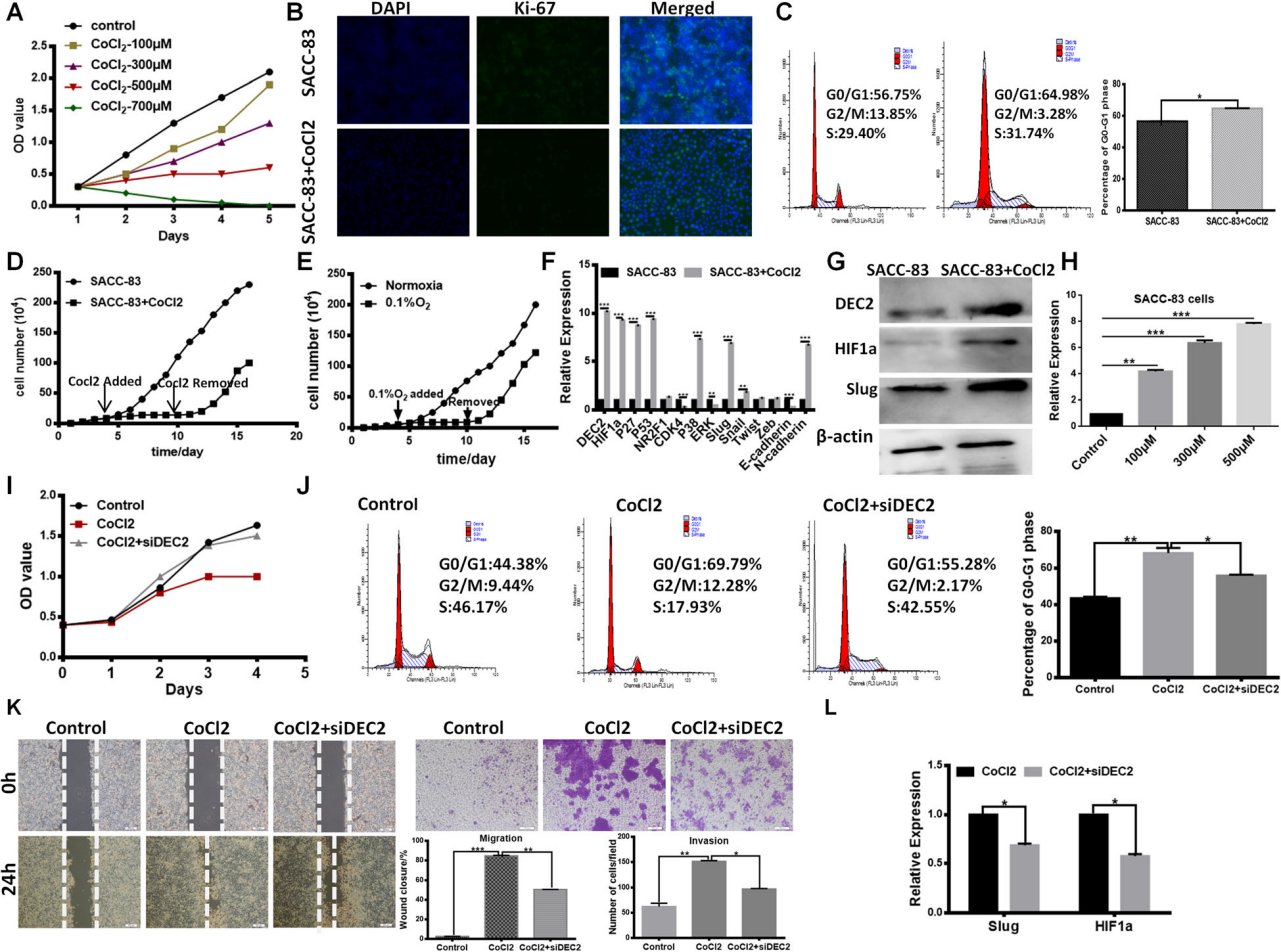
High expression of DEC2 was necessary for CoCl_2_- induced dormancy. **a** CCK-8 analysis of SACC-83 cells treated with 100, 300, 500 and 700 μM CoCl_2_. **b** and **c** The expression of Ki-67 and cell cycle of CoCl_2_ treated cells were determined by immunofluorescence and flow cytometry. **d** Cell growth analysis of SACC-83 cells treated with 500 μM CoCl_2_ for 7 days (from day 4 to day 10) and then recovered in normal media. **e** Cell growth analysis of SACC-83 cells treated with 0.1% O_2_ for 7 days (from day 4 to day 10) and then recovered into normoxia environment. **f** The expression of DEC2, HIF1α, P27, P53, NR2F1, CDK4, P38, ERK, Slug, Snail, Twist, Zeb, E-cadherin and N-cadherin were determined by QPCR in SACC-83/LM cells treated by CoCl_2_ and the control group. **g** The protein levels of DEC2, HIF1α and Slug in CoCl_2_ treatment and control SACC-83 cells. **h** DEC2 expression at different concentrations of CoCl_2_ in SACC-83. **i** and **j** Proliferation capacity and cell cycle were determined by CCK-8 assay (**i**) and flow cytometry (**j**). **k** Migration and invasion abilities of CoCl_2_ treatment and CoCl_2_ + siDEC2 SACC-83 cells were detected by Transwell and scratch assays, respectively. **l** The mRNA levels of Slug and HIF-1α in CoCl_2_ or CoCl_2_ + siDEC2 treatment SACC-83 cells. Error bars represent the mean ± SD of triplicate experiments (*P < 0 .05, **P < 0.01, ***P < 0 .001)

Many studies have displayed the close relationship between hypoxia and tumor dormancy [17–19], and here we have demonstrated that DEC2 was positively correlated with HIF1α expression in SACC cells. Hence, we proposed that due to the abundance of oxygen in the lung tissue, DEC2-overexpressed dormant cells transferred here are reactivated to form metastases. Here, we first found the proliferation of SACC-83 cells was suppressed in a dose-dependent manner up to 500 μM of CoCl_2_ (Fig. 4a). And the ratio of positive Ki-67 was markedly reduced (Fig. 4b) and cell population arrested in G0/G1 phase was increased in CoCl_2_ treated SACC-83 cells (Fig. 4c), indicating that a population of these hypoxic cells may enter into dormant state. Then, cell growth analysis suggested that while SACC-83 cells proliferation were suppressed during 7-day CoCl_2_ treatment, they recovered growth after removal of CoCl_2_ (Fig. 4d), demonstrating that growth-inhibited SACC-83 cells under CoCl_2_ treatment were dormant instead of senescent and apoptotic. Cell growth curves were similar between 0.1% hypoxia condition and CoCl_2_ treatment, indicating that tumor dormancy induced by CoCl_2_ is similar to true hypoxia. And cell proliferation was suppressed during 7-day 0.1% hypoxia condition, then resumed growth after changing to normoxia condition (Fig. 4e). Similar results were observed in SACC-LM cells (Additional file 1*:* Fig. S2A-F). This indicated that hypoxia under certain concentration may push cancer cells to enter into dormant state.

Furthermore, we found that the expression of DEC2, NR2F1, P53 and P27, hypoxia gene HIF1α and EMT transcription factor slug were all upregulated in SACC-83 cells treated by CoCl_2_ (Fig. 4f and g*)*. Interestingly, DEC2 expression presented dose-dependent increase up to 500uM of CoCl_2_, which indicated that DEC2 contributed to hypoxia-induced dormant process (Fig. 4h). The same results were displayed in SACC-LM cells (Additional file 1*:* Fig. S2G and H). We further silenced DEC2 using siRNA in CoCl_2_ treated SACC-83 cells and found that suppression of DEC2 resulted in reversible proliferation of dormant SACC-83 cells under CoCl_2_ treatment (Fig. 4i). Consistent with the reversible growth, cell population arrested in G0/G1 phase was reduced after DEC2 silencing during CoCl_2_ treatment in SACC cells (Fig. 4j). Furthermore, the expression of Ki-67 was also up-regulated (Additional file 1*:* Fig. S2I). Knockdown of DEC2 impeded cell invasiveness and metastatic potential increased by CoCl_2_ treatment (Fig. 4k), accompanying downregulation of Slug and HIF-1α (Fig. 4l). Thus, our loss-of-function study showed that the suppression of DEC2 could inhibit cell dormancy induced by CoCl_2_ treatment, indicating that different hypoxia states in primary and metastasis lesion may regulate DEC2- induced dormancy to lead to keep or reawaken cell dormancy state.

### Slug-induced EMT was involved in DEC2- induced dormancy

**Fig. 5:**
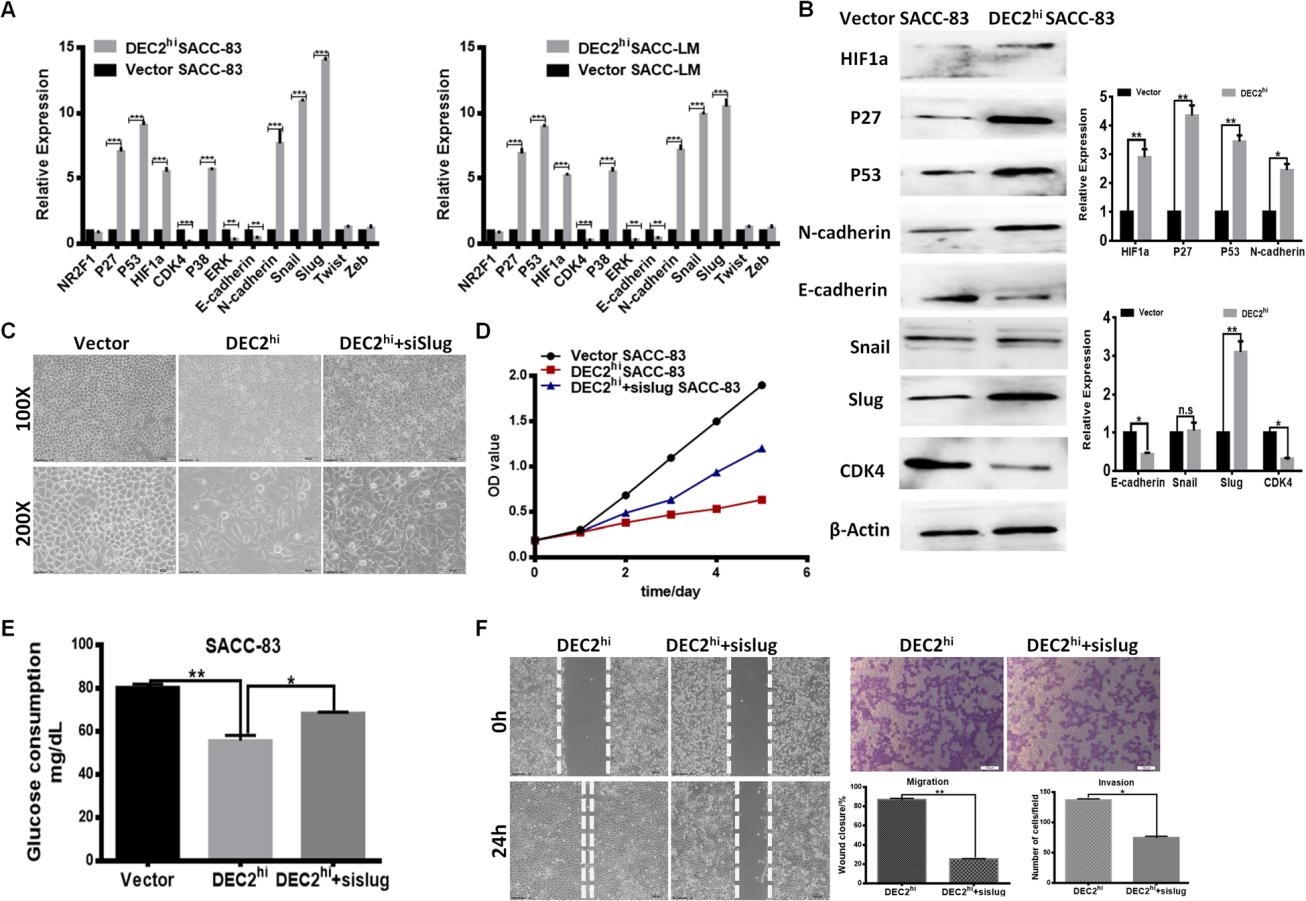
Slug-induced EMT was involved in DEC2- induced dormancy. **a** QPCR analyses of NR2F1, P27, P53, HIF1α, CDK4, P38, ERK, E-cadherin, N-cadherin, Snail, Slug, Twist and Zeb in DEC2 overexpressed and vector SACC-83/LM cells. **b** The protein expression of HIF1α, P27, P53, N-cadherin, Slug, Snail, CDK4 and E-cadherin were detected by western blot in DEC2 overexpressed and vector SACC-83 cells. **c** Cell morphologies were changed after Slug knockdown in DEC2 overexpressed SACC-83 cells. **d** and **e** Cell proliferation **(d)** and metabolism **(e)** were determined by CCK-8 assay and glucose consumption in DEC2^hi^/DEC2^hi^ + siSlug/vector SACC-83 cells. **f** Transwell chamber and scratch assays for DEC2^hi^ or DEC2^hi^ + siSlug SACC-83 cells. Error bars represent the mean ± SD of triplicate experiments (*P < 0 .05, **P < 0.01, ***P < 0 .001)

Recently, it was proposed that EMT positive tumor cells always characterized by low proliferation rate or quiescence and EMT program may have a potential role during tumor dormancy [20, 21]. We next analyzed the expressions of dormant related genes including NR2F1, P27, P53, HIF1α, CDK4, P38, ERK, E-cadherin, N-cadherin and EMT transcription factors (Snail/Slug/Twist/Zeb) in SACC-83 and SACC-LM cells with DEC2-overexpressed vector. The results showed that HIF1α, P27, P53, Snail, Slug and N-cadherin levels were significantly boosted after DEC2 overexpression, whereas CDK4 and E-cadherin were downregulated. But the protein expression of Snail did not change significantly. Moreover, the ERK/p38 signaling ratio was also decreased. NR2F1, Twist and Zeb were not significantly affected after DEC2 overexpression (Fig. 5a and b). We also silenced DEC2 in SACC-83 and SACC-LM cells and the opposite trend was observed (Additional file 1*:* Fig. S3). This showed that EMT had involved in the dormancy of SACC cells.

In order to further examine the role of EMT program during DEC2 induced tumor dormancy, we silenced Slug using siRNA in DEC2 overexpressed SACC-83 cells. The SACC-83 vector cells had an epithelial morphology, whereas DEC2-overexpression group exhibited a more spindle-cell morphology with tentacles. And suppression of Slug in DEC2 overexpressed cells reversed their spindle changes (Fig. 5c), proliferation capacity (Fig. 5d) and glucose consumption (Fig. 5e), indicating their dormant state was partial reversed. Additionally, downregulated Slug expression suppressed their migration and invasion significantly (Fig. 5f). These results showed that DEC2 may promote SACC dormancy by regulating EMT directly through Slug and thus enhance their migration and invasion ability.

### High expression of DEC2 associated with tumor dormancy in the primary SACC patients

**Fig. 6:**
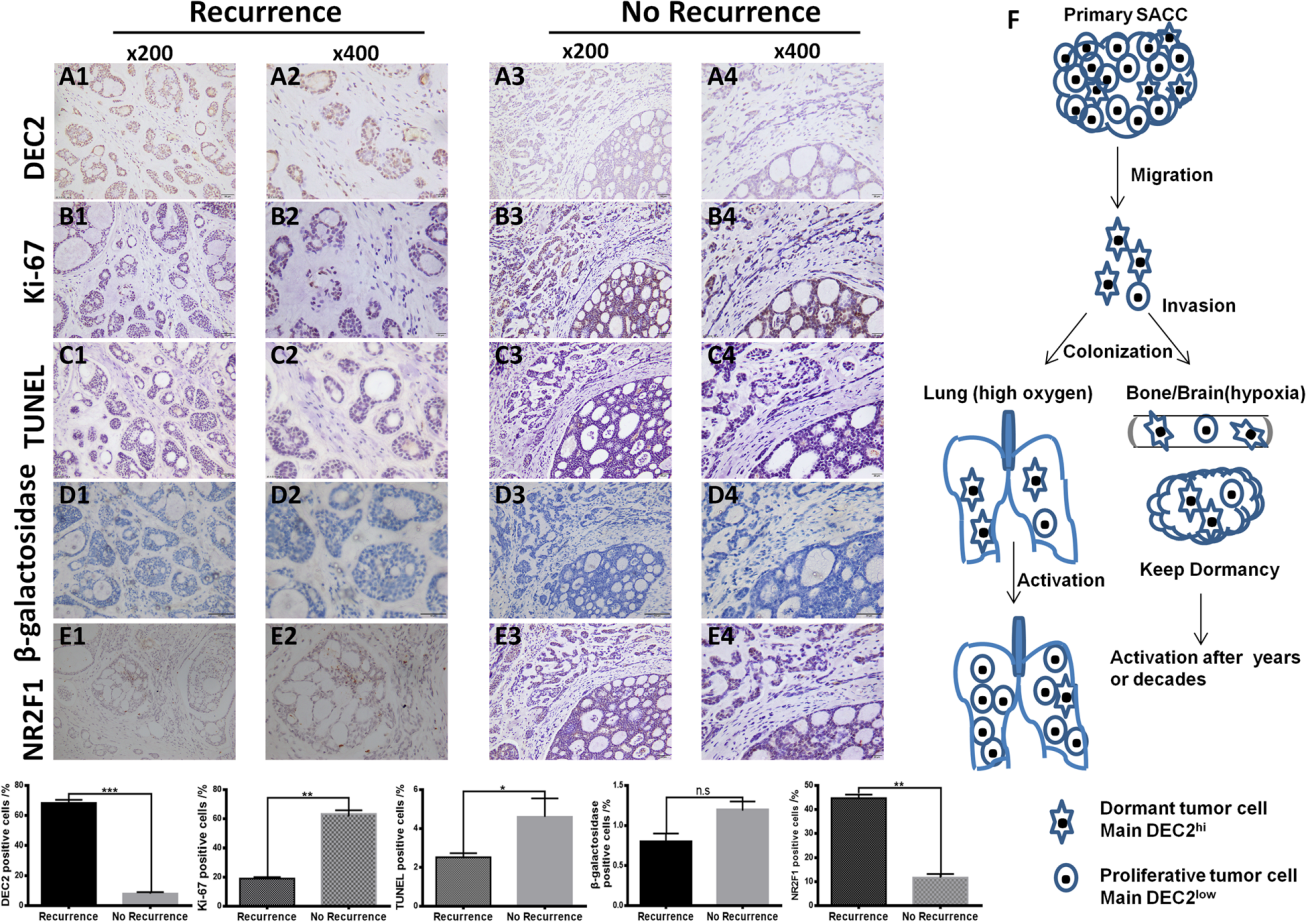
High expression of DEC2 associated with tumor dormancy in the primary SACC patients. **a** and **b** Expression levels of DEC2 and Ki-67 were analyzed by Immunohistochemical staining. **c** and **d** TUNEL and senescence tests of SACC between recurrence and no recurrence were shown. **e** Expression of NR2F1 was detected by Immunohistochemical staining. Student’s paired t test was used to analyze the differences between the metastasis and no metastasis specimens (*P < 0 .05, **P < 0.01, ***P < 0 .001). **s** Schematic figure illustrated dormancy during tumor progression and the functions of DEC2 in that (Round cell: Proliferative tumor cell, main DEC2^low^; Fusiform cells: Dormant tumor cell, main DEC2^hi^) [22–24]

To investigate the significance of DEC2 in human SACC cases, we used a cohort of *70* primary SACC samples obtained from clinical patients and 10 normal salivary gland samples. Immunohistochemistry results showed that the nuclear staining of DEC2 was detected in *17* of *70* (*24%)* in SACC and 7 out of 10 (70%) in normal salivary gland samples, respectively (Fig. 6a). Then, we focused on the cases with DEC2 positive expression, which occupied the small part of SACC patients. We found that 14 out of DEC2 positive specimens had low positive expression of Ki-67 (*5–10%*) *(*Fig. 6b*, P* < 0.01) and the negative of TUNEL *(*Fig. 6c*, P* < 0.05) and senescence tests *(*Fig. 6d, *P* > 0.05). In addition, SACC specimens were simultaneously immunostained for NR2F1, relevant to tumor dormancy. We found that DEC2 expression was significantly associated with NR2F1 (Fig. 6e, *P* < 0.001). This indicated that DEC2 positive tumor cells were neither proliferative nor apoptotic, which was consistent with the dormant phenotype of SACC patients.

We next investigated the expression of DEC2 and clinicopathologic parameters of SACC patients. The results showed that DEC2 was significantly associated with tumor site (*P* = 0.0438), histological subtype (*P* = 0.009), recurrence (*P* = 0.005) and metastasis (*P* = 0.002, Table 1*)*. But there was no significant association of DEC2 positive expression with age, gender and T stage. These suggested that positive expression of DEC2 in a few SACC cases was related to recurrence and metastasis of SACC patients, although there was negative expression of DEC2 in most of SACC patients. Schematic illustration was shown in Fig. 6f*.*

## Discussion

Tumor dormancy, mentioned in 1864 and described in 1954 by Hadfield as a temporary arrest in mitosis [25] has been defined as a clinical term [26]. It has been demonstrated that tumor dormancy was implicated in the invasion and metastasis of EMT program in many types of tumors. Here, we demonstrated that DEC2 participated in SACC dormancy under a high expression condition in xenograft of nude mice. And the formation of lung metastases was accompanied by low level of DEC2, which then reawakened dormancy and promoted proliferation of metastases. Then, we further addressed these different roles of DEC2 in primary and metastasis lesions and found that high expression of DEC2 involved in CoCl_2_-induced dormancy, indicating that different hypoxia states in primary and metastasis lesion may regulate DEC2-induced dormancy to keep or exit cell dormancy state. Further, overexpression of DEC2 induced the entry of SACC into dormancy, mediated by activating the expression of Slug, which then drived EMT program, contributing to growth arrest and dormancy, as well as enhanced migration and invasion capabilities. Our current finding of low level of DEC2 inducing SACC cells to exit dormancy in the second lesion reveals the role of DEC2 in regulating tumor cell dormancy has involved in hypoxia condition and EMT program.

DEC2 is one of the basic helix- loop-helix-Orange transcription factors, featured with a basic DNA binding domain, a helix-loop-helix (HLH) dimerization domain, and Orange extended dimerization domain [27]. And it has been implicated in regulating many types of biochemical processes, including circadian rhythm [28], cell proliferation and differentiation [29], apoptosis, hypoxia response, and EMT of tumor cells [28, 30]. It was demonstrated that DEC2 inhibited tumor cells proliferation in esophageal cancer and osteosarcoma [31, 32]. In this study, we demonstrated that DEC2 induced tumor dormancy of SACC both in vivo and vitro. It was supported by previous publication, which showed that the expression of DEC2 drived tumor dormancy in HNSCC and breast cancer [13, 33]. Our previous study has demonstrated that atRA treatment could also drive tumor dormancy of SACC by upregulating NR2F1 [15]. Sosa et al. proposed that NR2F1 was an important node in tumor dormancy induction and maintenance [34]. In the present study, we found that DEC2 was also participated in atRA induced dormancy. DEC2 was increased in atRA treated dormant cells and these cells were activated partially after DEC2 knockdown. But the expression changes of DEC2 did not affect NR2F1. The reason for this may be that this dormant process was mediated by complicated signal networks including DEC2 and NR2F1, but they did not regulate each other directly.

Hypoxia was a poor-prognosis microenvironmental feature of solid tumor, and Fluegen et al. proposed that primary tumor hypoxic microenvironment promoted the production of dormant tumor cells and resulted in chemo-radiotherapy resistance [16, 35]. It has also been demonstrated that hypoxic stress induced breast cancer dormancy, but the relationship and molecular mechanism within hypoxia and dormancy of SACC is still ambiguity [22]. Previous studies have shown that the expression of DEC2 and HIF1α were positively correlated during the progression of human osteosarcoma [36]. We found that high expression levels of HIF1α and P27 were all participated in the dormancy process regulated by DEC2 in SACC. And we also verified the validity of CoCl_2_ -based model in vitro for researching the relationship between tumor progression and hypoxic stress. We next detected the relationship between hypoxia and dormancy of SACC and the function of DEC2 during this process. The results showed that CoCl_2_ induced hypoxia–mimicking microenvironments can drive dormancy in SACC cells and high expression of DEC2 was necessary for the above dormant state. Furthermore, the dormant tumor cells could be reawakened when the microenvironment changed from hypoxia to normal oxygen. Consistent with the above results, we proposed that in mouse xenograft model, DEC2-overexpresed dormant SACC cells transferred into lung tissue and reactivated colony growth because of the abundant oxygen microenvironment. Exiting from dormancy always accompanied by DEC2 downregulation and resulted in the formation of numerous lung metastases and poor prognosis.

It has been proposed that EMT-positive cells may enter into dormant state [5, 20, 37], so we investigated the role of DEC2 in EMT inducing dormancy. In the present study, we initially found that DEC2 overexpressed dormant tumor cells displayed upregulation of Slug, one of the EMT transcription factors. Knockdown of Slug reversed their dormant state and suppressed migration and invasion. Therefore, we hypothesized that the clock gene DEC2 could drive tumor dormancy through inducing EMT program and thus promoting tumor cells ability of migration and invasion in SACC. Consistently, it was shown that tumor cells with the features of invasiveness which have undergone EMT always manifested characteristics of dormancy. And re-expression of E-cadherin always accompanied by proliferative activity which further indicated the critical roles of EMT during tumor dormancy [38]. Jiang et al. have also proposed that PRRX1 can drive the transition of EMT, and dormant state of cancer cells through miR-642b-3p in head and neck squamous cell carcinoma [39]. Therefore, cellular phenotype affects the process of tumor dormancy. Furthermore, it was reported that DEC2, one of the clock genes, promoted tumor metastasis and drived tumor dormancy [40]. To our knowledge, this is the first research suggesting that DEC2 induced EMT process to facilitate tumor dormancy through the control of Slug.

Additionally, we also investigated the expression of DEC2 in the primary SACC patients and normal salivary gland. The results showed that high expression of DEC2 was related with tumor dormancy and tumor site, histological subtype, and recurrence. We proposed the primary reason of poor prognosis caused by tumor dormancy is that dormant tumor cells are unstable and can resume proliferation as the external microenvironment changes. And just because of that, the purpose of tumor therapy might attempt to maintain the dormant state of tumor cells and prevent dormancy exit and growth resumption. Neophytou et al. proposed that the ultimate goal is to prolong the dormant period of metastatic tumor cells in breast cancer [41]. And it was also demonstrated that during tumor treatment, the Hippo signaling pathway contributes the initiation and stabilization of tumor dormancy [42]. But there are still questions that need to be answered to detect how DEC2 affect the growth state of tumor cells in different microenvironment.

## Conclusion

In this study, we showed two different dormancy fates of SACC cells by different expressions of DEC2 in the primary and metastasis of SACC. Several groups have reported that there are different oxygen concentrations in primary and metastasis tumors [43–45]. Our findings suggest the possibility that different hypoxia states in primary and metastasis lesion may regulate DEC2-induced dormancy to keep or awaken cell dormancy state. And DEC2 promoted the dormancy, EMT, migration and invasion of SACC cells, in which transcription factor Slug played an important role. A deep understanding of DEC2 function in tumor dormancy may provide a novel insight for improved treatment of SACC.

## Supplementary Information

**Additional file 1: Figure S1.** DEC2 induced dormancy of SACC-83 and SACC-LM cells. **A**: DEC2 overexpression inhibited proliferation and glucose consumption of SACC-LM cells. **B**: DEC2 overexpression increased cell population arrested in G0/G1 of SACC-LM cells. **C**: DEC2 did not change the proportion of apoptotic cells in SACC-LM cells. **D**: The mRNA and protein expression patterns of DEC2 silence in SACC-83 cells. **E and F**: Knockdown of DEC2 in SACC-83 and SACC-LM cells reversed their proliferation and glucose consumption. **Figure S2.** CoCl_2_ and hypoxia induced dormancy of SACC-LM cells. **A**: The cell growth curves of SACC-LM cells under different concentrations of CoCl_2_. **B**: CoCl_2_ treatment suppressed proliferation of SACC-LM cells for 16 days probably. **C**: Cell growth analysis of SACC-LM cells treated with 500 μM CoCl_2_ for 7 days (from day 4 to day 10) and then recovered in normal media. **D**: CoCl_2_ treatment inhibited glucose consumption of SACC-LM cells. **E**: Cell growth curves of SACC-LM cells induced by 0.1% O_2_ and 500 μM CoCl_2_. **F**: Cell growth analysis of SACC-LM cells treated with 0.1% O_2_ for 7 days (from day 4 to day 10) and then recovered into normoxia environment. **G**: The mRNA levels of DEC2, NR2F1, P53 and P27, HIF1α, P38/ERK and EMT related genes in SACC-LM cells treated by CoCl_2_. **H**: DEC2 expression of SACC-LM cells treated by different concentration of CoCl_2_. **I**: The expression of Ki-67 in SACC-83, SACC-83+ CoCl_2_ and SACC-83+ CoCl_2_ + siDEC2. **Figure S3.** The expression of dormant and EMT markers in SACC-83 and SACC-LM cells after DEC2 knockdown.

**Additional file 2: Supplementary Table 1**. Real-Time RT-PCR primer sequences.

## Data Availability

The datasets supporting the conclusions of this article are included within the article and its additional files.

## Abbreviations

SACC: Salivary adenoid cystic carcinoma
DEC: Differentiated embryonic chondrocyte gene
EMT: Epithelial-mesenchymal transition
NR2F1: Nuclear receptor subfamily 2 group F member 1
HIF: Hypoxia inducible factor
