# Supplementary figures and images for: Inhibition of DEC2 is necessary for exiting cell dormancy in salivary adenoid cystic carcinoma
Source: J Exp Clin Cancer Res. 2021 May 14;40:169. doi: 10.1186/s13046-021-01956-0 (PMC8120837; doi:10.1186/s13046-021-01956-0)

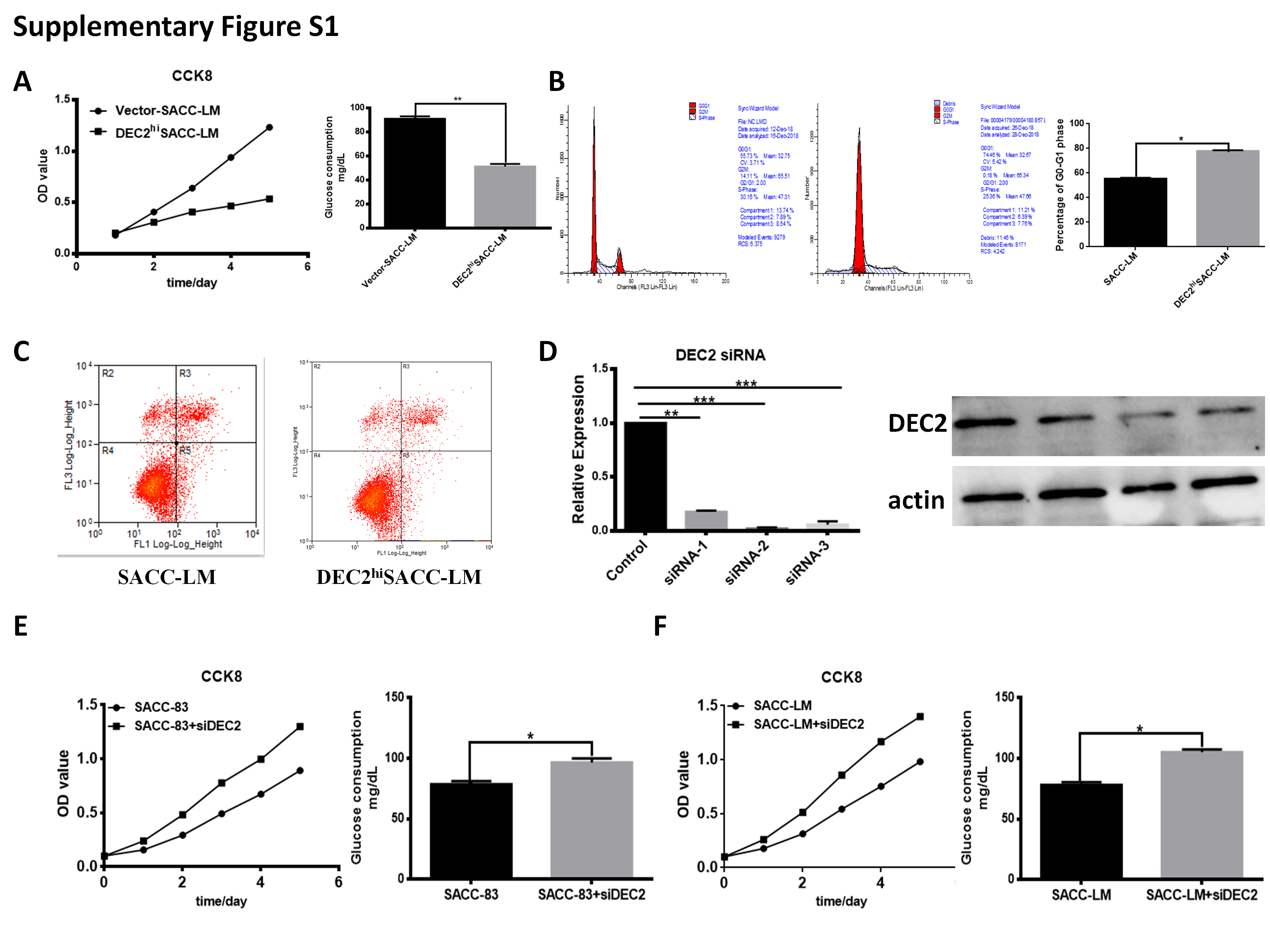

Supplement: Supplementary file 1 — Additional file 1: Figure S1. DEC2 induced dormancy of SACC-83 and SACC-LM cells. A: DEC2 overexpression inhibited proliferation and glucose consumption of SACC-LM cells. B: DEC2 overexpression increased cell population arrested in G0/G1 of SACC-LM cells. C: DEC2 did not change the proportion of apoptotic cells in SACC-LM cells. D: The mRNA and protein expression patterns of DEC2 silence in SACC-83 cells. E and F: Knockdown of DEC2 in SACC-83 and SACC-LM cells reversed their proliferation and glucose consumption. Figure S2. CoCl2 and hypoxia induced dormancy of SACC-LM cells. A: The cell growth curves of SACC-LM cells under different concentrations of CoCl2. B: CoCl2 treatment suppressed proliferation of SACC-LM cells for 16 days probably. C: Cell growth analysis of SACC-LM cells treated with 500 μM CoCl2 for 7 days (from day 4 to day 10) and then recovered in normal media. D: CoCl2 treatment inhibited glucose consumption of SACC-LM cells. E: Cell growth curves of SACC-LM cells induced by 0.1% O2 and 500 μM CoCl2. F: Cell growth analysis of SACC-LM cells treated with 0.1% O2 for 7 days (from day 4 to day 10) and then recovered into normoxia environment. G: The mRNA levels of DEC2, NR2F1, P53 and P27, HIF1α, P38/ERK and EMT related genes in SACC-LM cells treated by CoCl2. H: DEC2 expression of SACC-LM cells treated by different concentration of CoCl2. I: The expression of Ki-67 in SACC-83, SACC-83+ CoCl2 and SACC-83+ CoCl2 + siDEC2. Figure S3. The expression of dormant and EMT markers in SACC-83 and SACC-LM cells after DEC2 knockdown. [file 13046_2021_1956_MOESM1_ESM.zip › Figure S1.TIF]

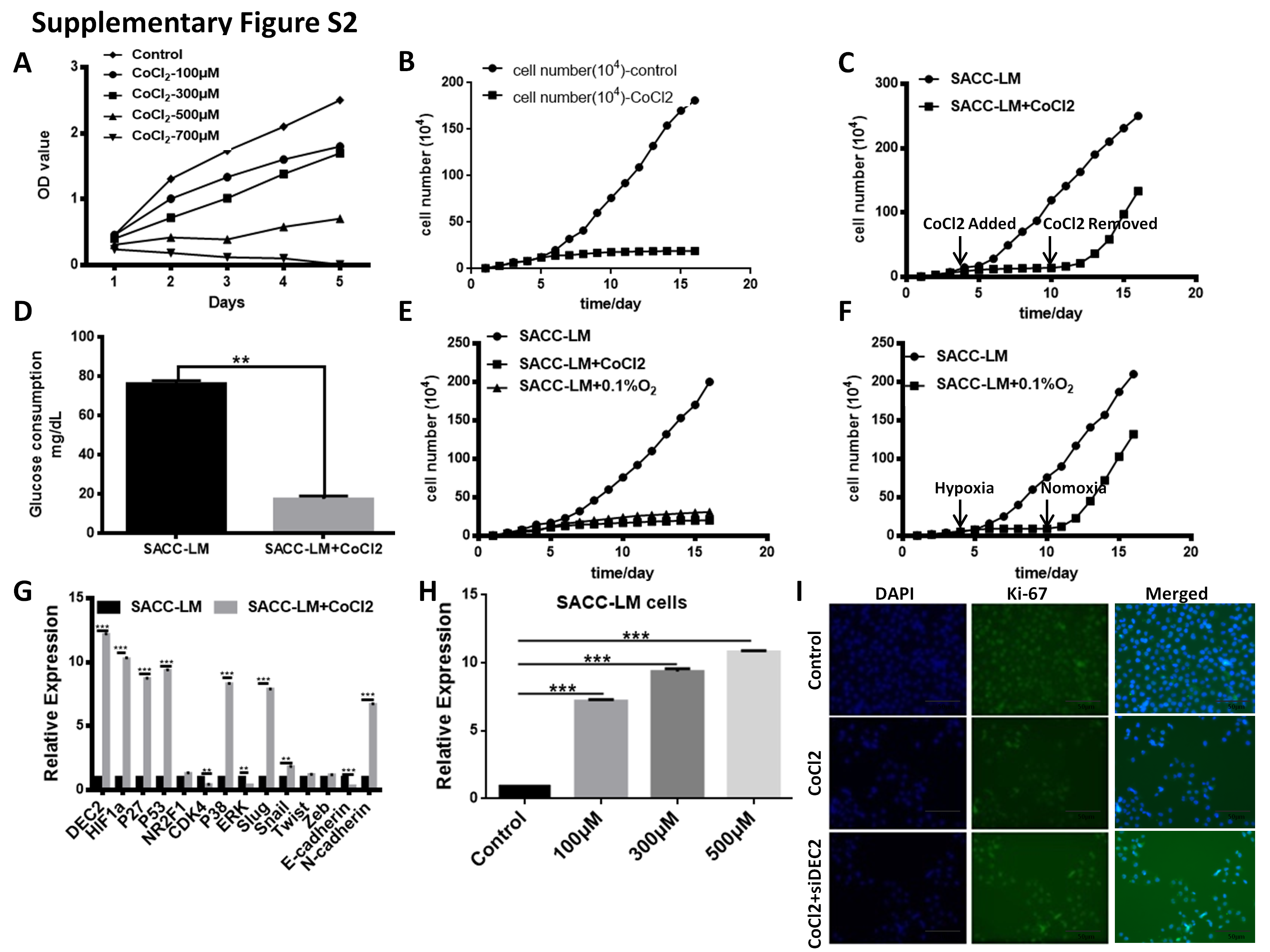

Supplement: Supplementary file 1 — Additional file 1: Figure S1. DEC2 induced dormancy of SACC-83 and SACC-LM cells. A: DEC2 overexpression inhibited proliferation and glucose consumption of SACC-LM cells. B: DEC2 overexpression increased cell population arrested in G0/G1 of SACC-LM cells. C: DEC2 did not change the proportion of apoptotic cells in SACC-LM cells. D: The mRNA and protein expression patterns of DEC2 silence in SACC-83 cells. E and F: Knockdown of DEC2 in SACC-83 and SACC-LM cells reversed their proliferation and glucose consumption. Figure S2. CoCl2 and hypoxia induced dormancy of SACC-LM cells. A: The cell growth curves of SACC-LM cells under different concentrations of CoCl2. B: CoCl2 treatment suppressed proliferation of SACC-LM cells for 16 days probably. C: Cell growth analysis of SACC-LM cells treated with 500 μM CoCl2 for 7 days (from day 4 to day 10) and then recovered in normal media. D: CoCl2 treatment inhibited glucose consumption of SACC-LM cells. E: Cell growth curves of SACC-LM cells induced by 0.1% O2 and 500 μM CoCl2. F: Cell growth analysis of SACC-LM cells treated with 0.1% O2 for 7 days (from day 4 to day 10) and then recovered into normoxia environment. G: The mRNA levels of DEC2, NR2F1, P53 and P27, HIF1α, P38/ERK and EMT related genes in SACC-LM cells treated by CoCl2. H: DEC2 expression of SACC-LM cells treated by different concentration of CoCl2. I: The expression of Ki-67 in SACC-83, SACC-83+ CoCl2 and SACC-83+ CoCl2 + siDEC2. Figure S3. The expression of dormant and EMT markers in SACC-83 and SACC-LM cells after DEC2 knockdown. [file 13046_2021_1956_MOESM1_ESM.zip › Figure S2.TIF]

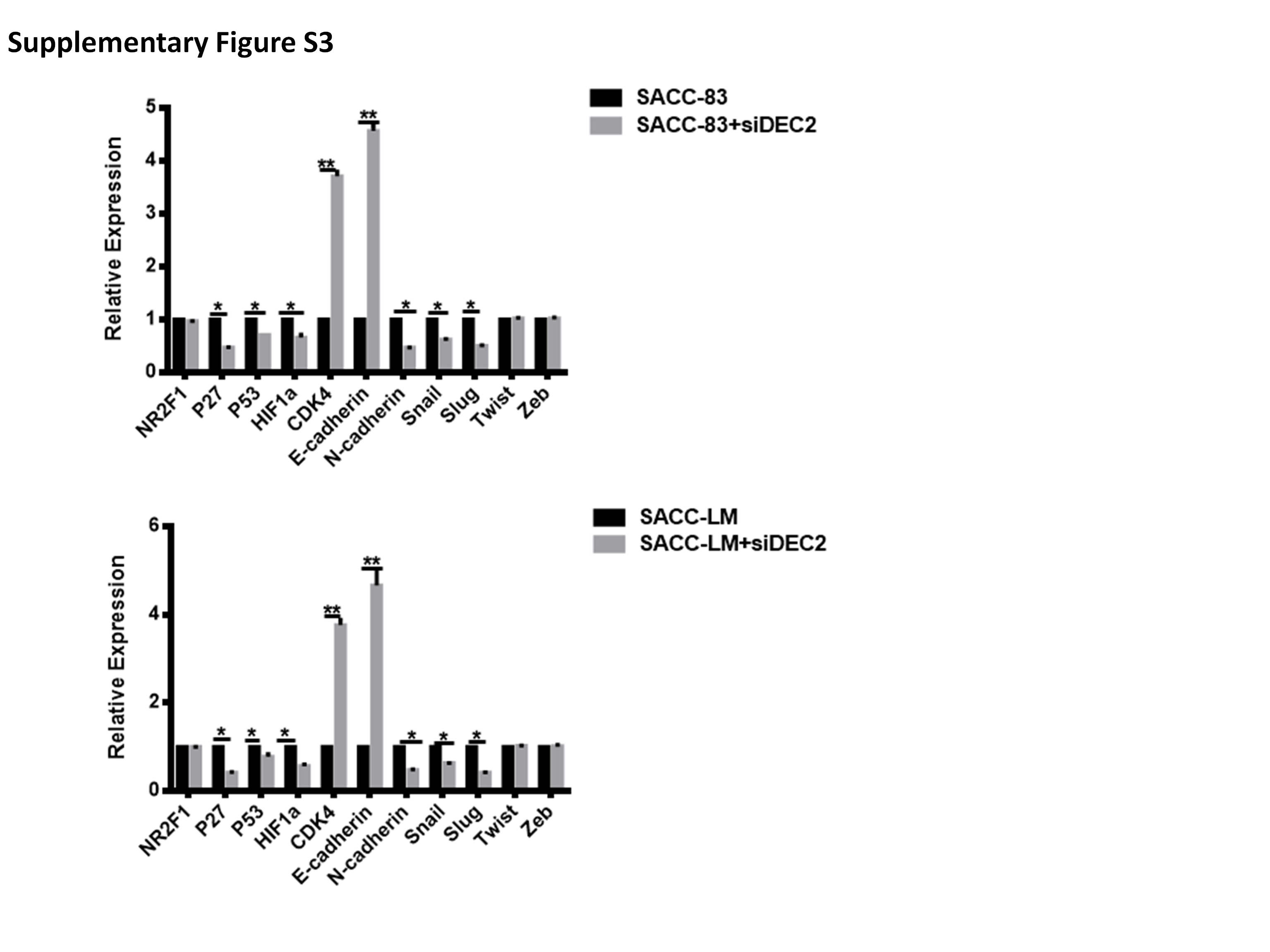

Supplement: Supplementary file 1 — Additional file 1: Figure S1. DEC2 induced dormancy of SACC-83 and SACC-LM cells. A: DEC2 overexpression inhibited proliferation and glucose consumption of SACC-LM cells. B: DEC2 overexpression increased cell population arrested in G0/G1 of SACC-LM cells. C: DEC2 did not change the proportion of apoptotic cells in SACC-LM cells. D: The mRNA and protein expression patterns of DEC2 silence in SACC-83 cells. E and F: Knockdown of DEC2 in SACC-83 and SACC-LM cells reversed their proliferation and glucose consumption. Figure S2. CoCl2 and hypoxia induced dormancy of SACC-LM cells. A: The cell growth curves of SACC-LM cells under different concentrations of CoCl2. B: CoCl2 treatment suppressed proliferation of SACC-LM cells for 16 days probably. C: Cell growth analysis of SACC-LM cells treated with 500 μM CoCl2 for 7 days (from day 4 to day 10) and then recovered in normal media. D: CoCl2 treatment inhibited glucose consumption of SACC-LM cells. E: Cell growth curves of SACC-LM cells induced by 0.1% O2 and 500 μM CoCl2. F: Cell growth analysis of SACC-LM cells treated with 0.1% O2 for 7 days (from day 4 to day 10) and then recovered into normoxia environment. G: The mRNA levels of DEC2, NR2F1, P53 and P27, HIF1α, P38/ERK and EMT related genes in SACC-LM cells treated by CoCl2. H: DEC2 expression of SACC-LM cells treated by different concentration of CoCl2. I: The expression of Ki-67 in SACC-83, SACC-83+ CoCl2 and SACC-83+ CoCl2 + siDEC2. Figure S3. The expression of dormant and EMT markers in SACC-83 and SACC-LM cells after DEC2 knockdown. [file 13046_2021_1956_MOESM1_ESM.zip › Figure S3.TIF]
