# Supplementary material for: Inhibition of DEC2 is necessary for exiting cell dormancy in salivary adenoid cystic carcinoma
Source: J Exp Clin Cancer Res. 2021 May 14;40:169. doi: 10.1186/s13046-021-01956-0 (PMC8120837; doi:10.1186/s13046-021-01956-0)
Supplement: Supplementary file 2 — Additional file 2: Supplementary Table 1. Real-Time RT-PCR primer sequences. [file 13046_2021_1956_MOESM2_ESM.docx]

Supplementary Table 1 Real-Time RT-PCR primer sequences.

| Primer | Forward sequence (5’ to 3’) | Reverse sequence (5’ to 3’) |
| --- | --- | --- |
| β-actin | GGCATCCTCACCCTGAAGTA | GGGGTGTTGAAGGTCTCAAA |
| DEC2 | CTGATGCTGTTGCTCGGTTA | TGCAGACTCTGGGACATCTG |
| NR2F1 | GCCTCAAAGCCATCGTGCTG | CCTCACGTACTCCTCCAGTG |
| P27 | ACGTGAGAGTGTCTAACGG | AGTGCTTCTCCAAGTC CC |
| Slug | AGATCTGCCAGACGCGAACT | GCATGCGCCAGGAATGTTCA |
| Twist | GTCCGCAGTCTTACGAGGAG | CCAGCTTGAGGGTCTGAATC |
| Snail | TCAAGATGCACATCCGAAGCC | TTGTGGAGCAGGGACATTCG |
| Zeb1 | GCACAACCAAGTGCAGAAGA | GCCTGGTTCAGGAGAAGATG |
| P53 | GCCCCTCCTCAGCATCTTATCCG | TCCCAGGACAGGCACAAACACGC |
| E-cadherin | TGCCCAGAAAATGAAAAAGG | GTGTATGTGGCAATGCGTTC |
| N-cadherin | ACAGTGGCCACCTACAAAGG | CCGAGATGGGGTTGATAATG |
| CDK4 | TGGAGCGTTGGCTGTATCTT | CAGTCGTCTTCTGGAGGCAA |
| P38 | GCATAATGGCCGAGCTGTTGACTGG | AAGGGCTTGGGCCGCTGTAATTCTCT |
| ERK | GGAGGACCTGAATTGTATCA | CTCCACTGTGATCCGTTTAT |
